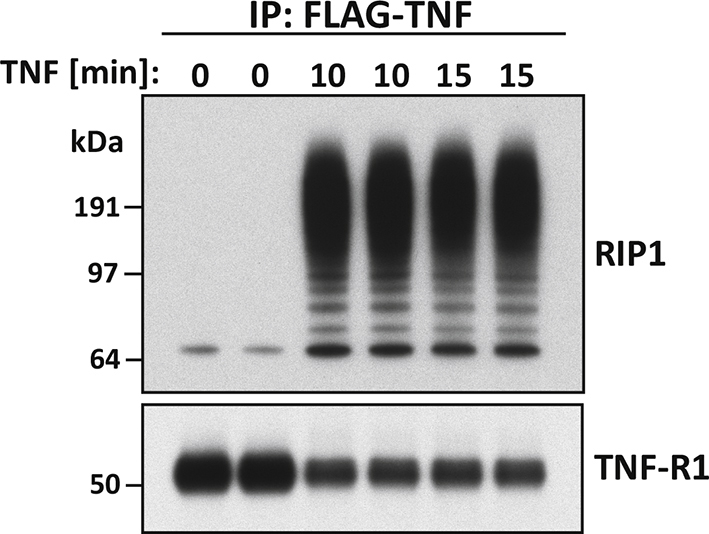# Optimising methods for the preservation, capture and identification of ubiquitin chains and ubiquitylated proteins by immunoblotting

**DOI:** 10.1016/j.bbrc.2015.08.109

**Published:** 2015-10-09

**Authors:** Christoph H. Emmerich, Philip Cohen

**Affiliations:** Medical Research Council Protein Phosphorylation and Ubiquitylation Unit, Sir James Black Centre, University of Dundee, Dundee DD1 5EH, Scotland, United Kingdom

**Keywords:** Ubiquitin, Ubiquitin-binding-domain, Immunoblotting, Deubiquitylase, Ubiquitin chain topology, DMP, dimethyl pimelidinate, DUB, deubiquitylase, GST, glutathione S-transferase, HEK, human embryonic kidney, IAA, iodoacetamide, IL-1R, IL-1 receptor, IMAC, immobilised metal ion affinity chromatography, IP, immunoprecipitation, IRAK, interleukin receptor associated kinase, LUBAC, Linear UBiquitin Assembly Complex, MES, 2-(N-morpholino) ethane sulfonic acid, MOPS, 3-(N-morpholino) propane sulfonic acid, NC, nitrocellulose, NEM, N-ethylmaleimide, NEMO, NF-κB essential modulator, NZF, Npl4 zinc finger, POI, protein of interest, PPase, phage λ-phosphatase, pUb, polyubiquitin, PVDF, polyvinyl difluoride, RIP, receptor interacting protein, SDS, sodium dodecyl sulphate, TA, tris-acetate, TG, tris-glycine, TNF, tumour necrosis factor, TNF-R, TNF receptor, TUBEs, tandem-repeated ubiquitin-binding entities, UBA, ubiquitin-associated, UBAN, ubiquitin binding in ABIN and NEMO domain, UBD, ubiquitin-binding domain, UIM, ubiquitin-interacting motif

## Abstract

Immunoblotting is a powerful technique for the semi-quantitative analysis of ubiquitylation events, and remains the most commonly used method to study this process due to its high specificity, speed, sensitivity and relatively low cost. However, the ubiquitylation of proteins is complex and, when the analysis is performed in an inappropriate manner, it can lead to the misinterpretation of results and to erroneous conclusions being reached. Here we discuss the advantages and disadvantages of the methods currently in use to analyse ubiquitin chains and protein ubiquitylation, and describe the procedures that we have found to be most useful for optimising the quality and reliability of the data that we have generated. We also highlight commonly encountered problems and the pitfalls inherent in some of these methods. Finally, we introduce a set of recommendations to help researchers obtain high quality data, especially those new to the field of ubiquitin signalling. The specific topics addressed in this article include sample preparation, the separation, detection and identification of particular ubiquitin chains by immunoblotting, and the analysis of ubiquitin chain topology through the combined use of ubiquitin-binding proteins and ubiquitin linkage-specific deubiquitylases.

## 1 Introduction

**Fig. 1:**
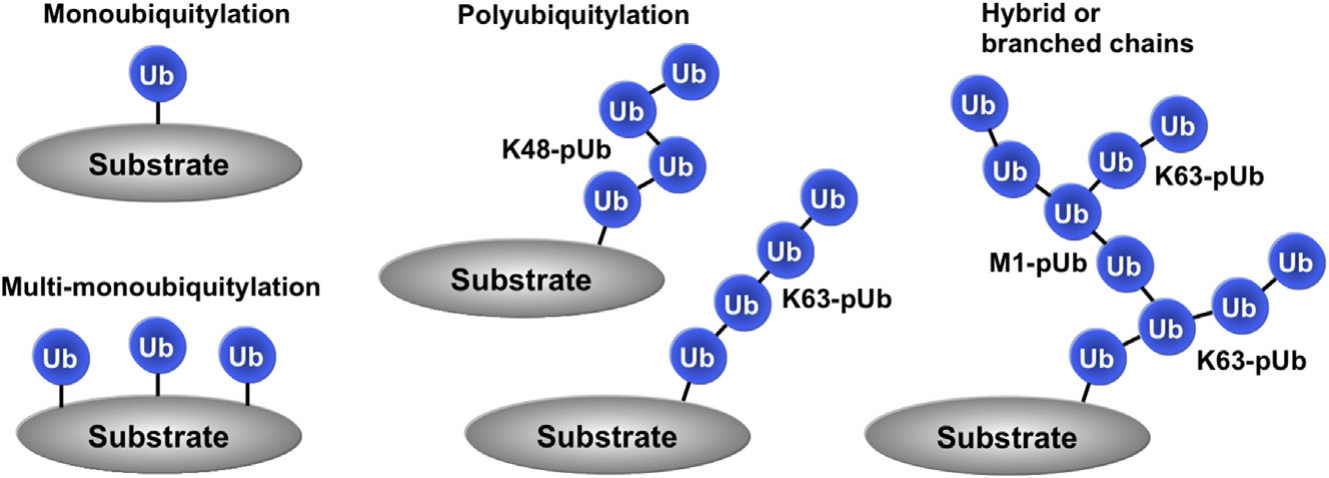
Different types of ubiquitylation. Ubiquitin modifications can be classified into three general types, termed monoubiquitylation, multi-monoubiquitylation and polyubiquitylation. Polyubiquitylation can be further subdivided into homotypic ubiquitylation (each ubiquitin chain comprising just one type of ubiquitin linkage) or heterotypic ubiquitylation (containing more than one type of ubiquitin chain). The latter are usually termed hybrid, branched or mixed ubiquitin chains.

The discovery of ubiquitin-mediated proteolysis [1] was one of the most seminal papers published in *Biochemical and Biophysical Research Communications* (BBRC), which won Aaron Ciechanover and Avram Herschko the Nobel Prize for Chemistry 26 years later. Subsequently, ubiquitylation was found to control many other cellular processes and, to date, eight different types of ubiquitin chain linkage have been identified in cells [2], [3]. These linkages are formed by the covalent attachment of the C-terminus of ubiquitin to the ε-amino groups of any of the seven lysine (K) residues in ubiquitin (K6, K11, K27, K29, K33, K48 and K63) or the α-amino group of its N-terminal methionine (M1) residue. In addition, some proteins become mono-ubiquitylated or multi-monoubiquitylated, in which the first ubiquitin attached to a protein does not undergo polyubiquitylation. Finally, hybrid (also called branched or mixed) ubiquitin chains containing more than one type of ubiquitin linkage have also been identified in cells [4], [5], [6], introducing further complexity into the system (Fig. 1). Protein ubiquitylation is a versatile and reversible protein modification with regulatory roles that extend far beyond the proteasome-dependent degradation of substrate proteins, and include cellular signalling and trafficking, as well as the control of the cell division cycle and DNA repair.

In recent years there has been an explosion of interest in ubiquitylation and the number of publications in this area is increasing exponentially (Fig. S1). It is self evident that the experiments aimed at enhancing our understanding of this process are conducted to the highest standards of quality control. However, to our knowledge, no simple, clear guidelines or standardised methodologies for the preservation, detection and analysis of ubiquitylation events by immunoblotting are available. In this article, we therefore introduce a number of recommendations about how to optimise the quality of the information that can be obtained from such experiments, based on our own experiences and other published papers in the literature.

## 2 Preserving the ubiquitylation state of proteins

### 2.1 Inhibition of deubiquitylases

**Fig. 2:**
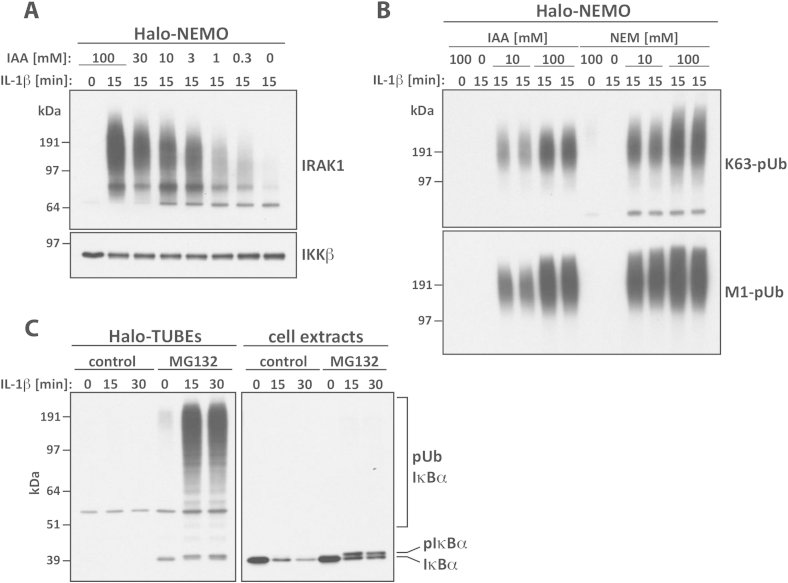
Importance of composition of lysis buffer to prevent deubiquitylation. (A) High concentrations of IAA are required to inactivate deubiquitylases and maintain the level of pUb chains in cell lysates. Human embryonic kidney (HEK) 293 cells stably expressing the IL-1 receptor (IL-1R cells) were stimulated for 15 min with 5 ng/ml IL-1β and lysed without or with the indicated concentrations of IAA. Cell lysates were incubated for 12 h with immobilised Halo-NEMO and the ubiquitylated forms of IRAK1 captured were identified by immunoblotting. Antibodies against IKKβ, which interacts with NEMO in a ubiquitin-independent manner, were used as a loading control. (B) As in A, except that IL-1R cells were lysed without or with 10 or 100 mM IAA or NEM, respectively. Ubiquitin chains were visualised by immunoblotting with antibodies that recognise K63-pUb or M1-pUb chains. (C) The proteasomal inhibitor MG132 can preserve the ubiquitylated forms of some POIs. IL-1R cells were incubated for 1 h without (control) or with 25 μM MG132 and then stimulated with 5 ng/ml IL-1β for the times indicated. Ubiquitylated IκBα captured from cell lysates using immobilised Halo-TUBEs was identified by immunoblotting (left hand panel). Cell extracts (20 μg protein) were also immunoblotted for IκBα (right hand panel). It should be noted that IL-1 induces the phosphorylation of IκBα (pIκBα), which leads to its K48-linked ubiquitylation and proteasomal destruction.

Protein ubiquitylation is reversible and this modification can therefore easily be lost through the hydrolysis of ubiquitin chain linkages, which is catalysed by protein ubiquitin hydrolases, termed deubiquitylases (DUBs). For this reason it is essential to include DUB inhibitors in the buffers used for cell lysis, to preserve proteins in the state of ubiquitylation at which they were present in the intact cell. The inclusion of DUB inhibitors is particularly critical during immunoprecipitation (IP) or other “pull-down” experiments, where cell extracts may be incubated for several hours in non-denaturing conditions. There are five different families of DUBs, one of which encodes metallo-proteinases, the other four being cysteine proteinases. Therefore, to block DUB activity, EDTA or EGTA must be included in the lysis buffer to remove traces of heavy metal ions, and Iodoacetamide (IAA) or N-ethylmaleimide (NEM) must also be added to alkylate the active site cysteine residues of DUBs. Although IAA or NEM have typically been included at concentrations of 5–10 mM in many publications, we find that up to 10-fold higher concentrations are needed to preserve the ubiquitylation status of some proteins (e.g. Interleukin receptor associated kinase-1 (IRAK1) (Fig. 2A) and ubiquitin chains (Fig. 2B). High concentrations of NEM are better at preserving K63-Ub chains and M1-Ub chains than high concentrations of IAA, probably due to the instability of the latter compound.

An advantage of IAA over NEM is that it is destroyed by light within minutes, preventing the continued alkylation of cysteine residues on many proteins. However, the covalent 2-acetamidoacetamide adduct (C_4_H_6_N_2_O_2_) formed by the reaction of IAA with cysteine residues has a molecular mass of 114 Da [7], which is identical to that of the Gly–Gly dipeptide that remains attached to the ε-amino group of lysine residues of proteins after ubiquitylated proteins have been digested with trypsin. This modification may therefore interfere with the identification of ubiquitylation sites by mass spectrometry. It is therefore recommended to use NEM instead of IAA when such mass spectrometry experiments are to be performed. NEM and IAA are equally compatible for experiments where immunoblotting is the final readout.

If the ubiquitylation of proteins is to be studied in cell extracts only, then DUBs can be inactivated by extracting the cells directly into boiling lysis buffer containing 1% sodium dodecyl sulphate (SDS). Ubiquitin with a C-terminal cysteine-reactive probe is reported to inactivate some DUBs [8] and, provided that this compound inhibits every DUB, it may also be useful for preventing deubiquitylation after cell lysis. Other broad-spectrum chemical inhibitors of DUBs may be identified in the future.

### 2.2 Proteasome inhibition

Proteins modified by all types of ubiquitin linkage, except for K63-linked and M1-linked chains, can be targeted to the 26S Proteasome for rapid degradation. For example, in yeast, proteins modified by K6-, K11-, K27-, K29- and K33-linked polyubiquitin (pUb) chains, as well as by K48-linked ubiquitin chains, accumulated in cells when the proteasome was inhibited [9]. Many inhibitors of the chymotryptic like protease of the proteasome have been described [10], of which the most widely used is Z-leucyl-leucyl-leucyl-CHO, termed MG132. Treatment with MG132 blocks protein degradation and preserves the ubiquitylated form of the protein of interest (POI), thereby facilitating its detection. As an example, pUb-IκBα was only detectable by immunoblotting if the cells were incubated with MG132 prior to cell lysis and enriched from the cell extracts using immobilised Halo-TUBEs (Tandem-repeated Ubiquitin-Binding Entities) (Fig. 2B, left panel), which capture every type of ubiquitin chain (Section 5). However, prolonged (12–24 h) treatment with MG132 can have cytotoxic effects [11] and ubiquitylation observed after these long incubations might be a consequence of one or more stress responses.

## 3 Resolution and identification of ubiquitin chains and ubiquitylated proteins by SDS-PAGE

### 3.1 Choice of gel and running buffer

**Fig. 3:**
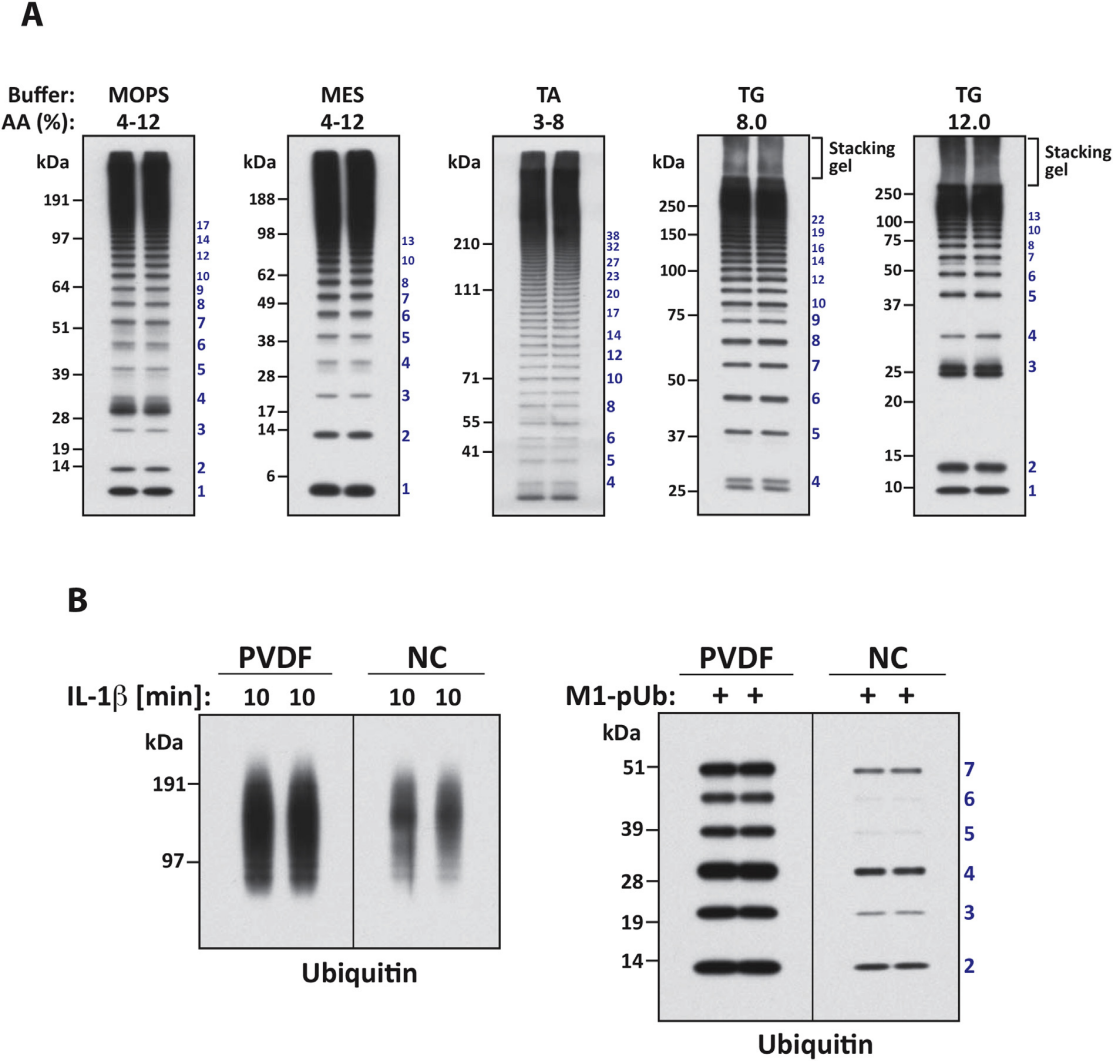
Importance of the buffer composition, polyacrylamide gel electrophoresis system and transfer conditions to optimise the detection of ubiquitin chains by immunoblotting. (A) The Linear UBiquitin Assembly Complex (LUBAC) was immunoprecipitated from cell extracts with antibodies raised against the catalytic component HOIP and incubated with UBE1, UBE2L3, ubiquitin and MgATP to generate M1-Ub chains, which were then separated using pre-cast NuPAGE Novex (4–12% acrylamide gradient) Bis-Tris gels with either MES-SDS or MOPS-SDS Running Buffer; or with pre-cast NuPAGE Novex (3–8% acrylamide gradient) Tris-Acetate (TA) gels and TA Running Buffer or 8% and 12% acrylamide gels prepared in our laboratory with a Tris-Glycine (TG) buffer. (B) M1-pUb chains present in the extracts of IL-1 stimulated IL-1R cells were captured on Halo-NEMO beads and subjected to SDS-PAGE and transferred either to a PVDF or a nitrocellulose (NC) membrane. M1-pUb chains (2–7 ubiquitin units, 25 ng) were included as a control. In A and B, immunoblotting was carried out with an anti-ubiquitin antibody (Dako). The numbers on the right hand side of each figure indicate the number of ubiquitin oligomers in each protein band.

Different gels and running buffers for resolving ubiquitylated proteins by SDS-PAGE are available. Proteins can be modified by 20 or more ubiquitin molecules that can add >200 kDa to their molecular mass, resulting in a smear of pUb chains that typically stretch upwards towards the top of the gel, so that selection of the most appropriate separation system is important (see Section 10). The action of E1, E2 and E3 ligases can generate pUb chains *in vitro* that differ greatly in length (Fig. 3A). When using pre-poured gradient gels, a MES (2-(N-morpholino) ethane sulfonic acid) buffer gives improved resolution of relatively small ubiquitin oligomers comprising 2–5 ubiquitins, whereas a MOPS (3-(N-morpholino) propane sulfonic acid) buffer gives improved resolution of pUb chains containing eight or more ubiquitins (Fig. 3A). On the other hand, a Tris-acetate (TA) buffer is superior for the resolution of proteins in the molecular mass range of 40–400 kDa (Fig. 3A). When using gels of a single acrylamide concentration of around 8% and a Tris-glycine (TG) buffer, it is still possible to separate individual ubiquitin chains comprising up to 20 ubiquitins (Fig. 3A). However, to be able to detect mono-ubiquitin and short ubiquitin oligomers, the acrylamide concentration has to be increased to around 12% (Fig. 3A), at the expense of reducing the resolution/separation of longer pUb chains.

### 3.2 Transfer from gels to membranes

To achieve complete transfer of the high molecular mass pUb chains separated by SDS-PAGE, we recommend electrophoretic transfer for 2.5 h at 30 V (see Supplementary Information for technical details). Faster transfer at higher voltages might prevent ubiquitin chains from re-folding correctly on the membrane, which can result in non-specific linkage recognition by pUb chain-specific antibodies [12]. Nitrocellulose (NC) and polyvinyl difluoride (PVDF) membranes have been widely used for the detection of ubiquitin chains. However, until now, no detailed comparison has been made between the two systems. In Fig. 3B, samples containing either longer or shorter M1-linked ubiquitin chains of defined length, were transferred to either PVDF (Millipore; pore size: 0.45 μm; capacity: 294 IgG/cm^2^) or nitrocellulose (Amersham/GE Healthcare; pore size: 0.45 μm; capacity: 115–125 IgG/cm^2^) membranes under identical conditions (see Supplementary Information for technical details) and immunoblotted in parallel with an anti-ubiquitin antibody. The specific signal intensity for ubiquitin was higher when PVDF membranes were used. Both, PVDF and nitrocellulose membrane systems, are also available with a pore size of 0.2 μm, which can be beneficial when analysing short ubiquitin chains [12].

## 4 Detection of ubiquitin and ubiquitin chains by immunoblotting

### 4.1 Enhancing the performance of anti-ubiquitin antibodies

Ubiquitin is a small globular protein that is difficult to denature and the ubiquitin epitopes might not be accessible to antibodies due to insufficient denaturation during SDS-PAGE. Conversely, if an anti-ubiquitin antibody is raised against the denatured protein, it may fail to recognise its epitope in native ubiquitin. Therefore, after transfer to PVDF or nitrocellulose membranes, the signal strength of anti-ubiquitin antibodies can frequently be enhanced significantly if the membrane is subjected to a denaturing treatment prior to blocking the primary antibody. Any one of the following methods can be used: 1. Incubation for 15–30 min in boiling water; 2. Incubation for 30 min at 4 °C in 20 mM Tris/HCl, pH 7.5, 5 mM 2-mercaptoethanol containing 6 M Guanidine/HCl; 3. Autoclaving [13].

### 4.2 Choosing the correct ubiquitin antibody

**Fig. 4:**
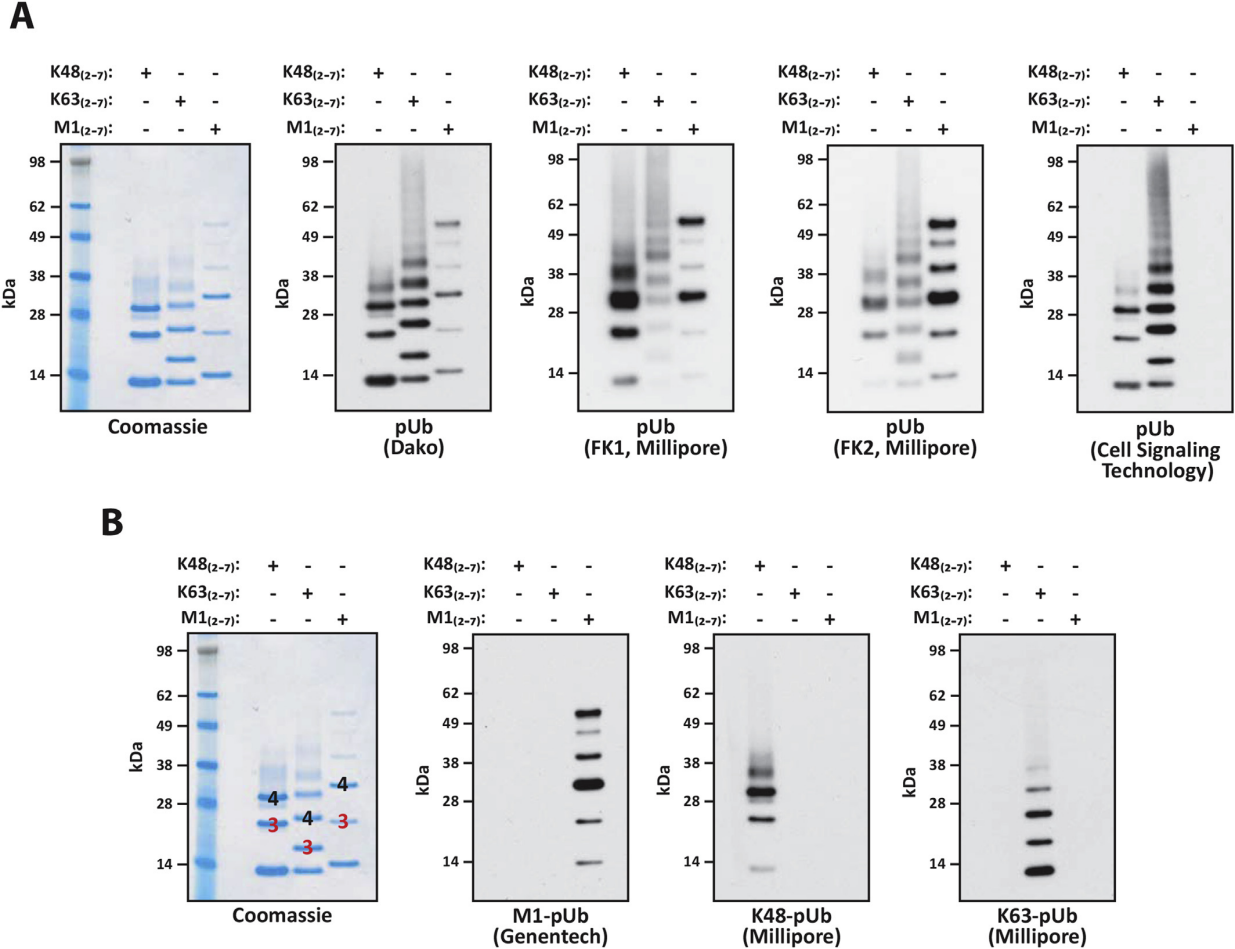
Importance of selecting the correct anti-ubiquitin antibody. (A) 25 ng of K48-linked (K48_2-7_), K63-linked (K63_2-7_) or M1-linked (M12-7) ubiquitin oligomers were separated by SDS-PAGE and visualised by immunoblotting with the different anti-ubiquitin antibodies indicated (the Coomassie-Blue stained gel is shown as a loading control). (B) As in A, except that K48-, K63- and M1-linked ubiquitin chains were detected with ubiquitin chain-specific antibodies. For comparison, Coomassie-stained K48-, K63- and M1-linked trimers and tetramers were labelled.

Many monoclonal or polyclonal antibodies that recognise ubiquitin are available commercially. Most of these bind both mono-ubiquitin and pUb chains attached covalently to substrate proteins, but antibodies that only recognise pUb chains and do not recognise monoubiquitin or mono-ubiquitylated proteins have also been developed [14]. What is not well known is that different anti-ubiquitin antibodies do not have equal affinities for all ubiquitin linkage types. For example, the anti-Ub antibody from Dako does not recognise M1-pUb oligomers as well as K48- and K63-pUb chains, the FK1 antibody binds preferentially to K48-linked oligomers and the FK2 antibody binds preferentially to M1-pUb oligomers compared to K48-Ub or K63-Ub oligomers (Fig. 4A). Interestingly, the anti-Ub antibody from Cell Signalling Technology barely recognises M1-Ub oligomers (Fig. 4A, right hand panel). Therefore, the selection of which anti-ubiquitin antibody to use can be critical.

### 4.3 Detection of different pUb chains by linkage-type-specific antibodies or by their electrophoretic mobility

At the time of writing, pUb chain-specific antibodies for four different chain types (K11-, K48-, K63- and M1-linked ubiquitin chains) are available commercially (e.g. Fig. 4B). The methods used to make these antibodies have been reviewed [15], [16], [17]. Although chemically identical, the electrophoretic mobility of small ubiquitin oligomers varies with linkage type. A simple and elegant method for distinguishing between different ubiquitin chain types is therefore to compare their relative rates of migration after SDS-PAGE (Fig. 4B) [5], [18]. The way in which this has been used to reveal the presence of hybrid ubiquitin chains is described in Section 6.2.

## 5 Capturing pUb chains and ubiquitylated proteins on immobilised ubiquitin-binding domains

An unambiguous way to demonstrate the covalent attachment of pUb chains to proteins is to capture the ubiquitylated form of the POI from cell extracts using immobilised ubiquitin-binding domains (UBDs) of defined ubiquitin chain specificity (Table 1) followed by immunoblotting with antibodies raised against the POI. A variety of ubiquitin receptors exist, which contain one or several UBDs that interact with mono-ubiquitin or different types of pUb chains. More than 20 different families of UBDs have been described so far [19], [20].

**Table 1:** Specificity of UBDs used to capture pUb chains and pUb-proteins.

Protein/Peptide	UBD	Specificity	Reference
NEMO	UBAN domain	M1 > K63 chains	[21], [22], [23]
NEMO [D311N]	UBAN domain	No Ub-binding	[21], [24], [25]
TAB2	NZF domain	K63 chains	[26]
TAB2 [T674A/F675A]	NZF domain	No Ub-binding	[27]
Ubiquilin-1	UBA domain	All Ub chains	[28]
MultiDsk	UBA domain	All Ub chains	[29]
RAP80	dual UIM domains	K63 chains	[30]
TRABID	NZF domain	K29 and K33 chains	[5]

**Fig. 5:**
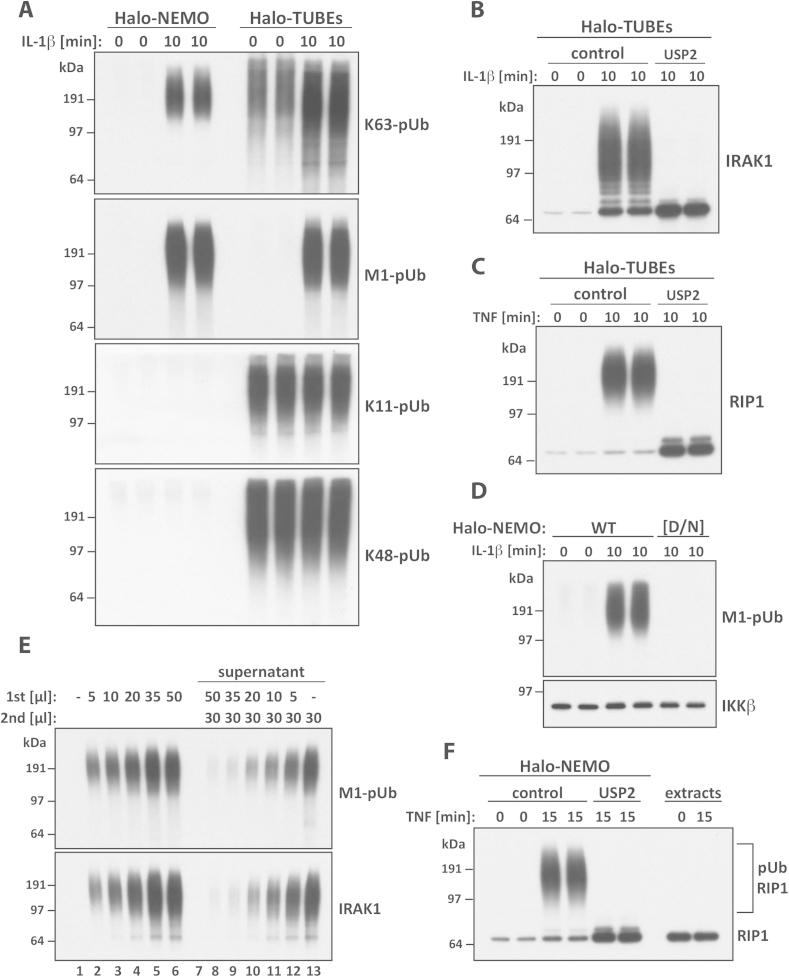
Characterisation of the Halo-UBD pull-down system. (A, B) IL-1R cells were stimulated for the times indicated with 5 ng/ml IL-1β, lysed and the pUb chains captured from the cell extracts using immobilised Halo-TUBEs or Halo-NEMO. The pUb chains released with SDS were subjected to SDS-PAGE and immunoblotted with the indicated ubiquitin chain-specific antibodies (A), or an IRAK1 antibody (B) after incubation of the Halo-TUBEs with λ-PPase in the absence (control) or presence of USP2 (B). (C) As in (B), except that RIP1 was captured from TNF-stimulated THP-1 cells (10 ng/ml for 10 min). Anti-RIP1 was used for immunoblotting. (D) As in A, except that M1-linked-pUb chains were captured using immobilised Halo-NEMO. The ubiquitin-binding-defective mutant Halo-NEMO[D311N] was included as a control. IKKβ, which interacts with NEMO in a ubiquitin-independent manner, was used as a loading control. (E) pUb chains and pUb-proteins were captured from 3 mg of cell extract protein using the indicated amounts of Halo-NEMO beads (Lanes 2–6) or Halo-beads (Lane 1), then released from the beads in SDS and identified by immunoblotting with antibodies against M1-pUb chains or IRAK1. In lanes 8–13, the supernatants from the pull-downs in lanes 1–6 were incubated with fresh Halo-NEMO beads and captured proteins were examined as before. Nearly all the M1-pUb chains and pUb-IRAK1 were removed from the cell extracts during the initial incubation, provided that ≥35 μl of Halo-NEMO beads were used (Lanes 5/6 and 8/9). (F) As in (C), except that THP-1 cells were stimulated for 15 min with 10 ng/ml TNF and RIP1 was captured from the cell extracts with Halo-NEMO. Cell extracts (20 μg protein) were subjected to SDS-PAGE. RIP1 was visualised by immunoblotting with an anti-RIP1 antibody.

When immobilised on a solid support, UBDs can be exploited to capture and so enrich pUb chains of different linkage types (Fig. 5A and Table 1) and proteins to which they are attached covalently (Fig. 5B and C) and non-covalently. If the pUb chains attached to the POI are of unknown topology, immobilised UBDs that can capture all types of ubiquitin chain should be used initially (Table 1), such as Halo-tagged TUBEs, (Fig. 5A), which consists of tandem UBA domain repeats of the protein Ubiquilin-1 (Fig. S2). For example, IRAK1 (Fig. 5B) and RIP1 (Receptor Interacting Protein 1) (Fig. 5C) undergo ubiquitylation within minutes when cells are stimulated with IL-1 and TNF (Tumour Necrosis Factor), respectively, and the ubiquitylated proteins can be captured by Halo-TUBEs.

To prove that material migrating more slowly than the POI is really caused by its ubiquitylation, the captured proteins must be treated with a non-specific DUB, such as USP2 [31], to reconvert the POI to the unmodified species (Fig. 5B and C). Combined treatment with a DUB and a phosphatase may be needed to regenerate the unmodified protein, since many proteins contain both types of covalent modification. Once the type(s) of pUb chains is (are) known more selective UBDs or UBD-containing proteins (Table 1) that discriminate between pUb chains can be used, such as NF-*κ*B essential modulator (NEMO) (Fig. 5A).

In these experiments, it is important to also employ an immobilised ubiquitin-binding-defective mutant of the UBD, to establish that the interaction with the ubiquitin chain or ubiquitylated protein is specific (Table 1 and Fig. 5D) and to distinguish these proteins from those that bind in an ubiquitin-independent manner. However, proteins that bind non-covalently to the UBD or UBD-containing protein in a stimulus- and ubiquitin-independent manner can be employed as loading controls in these experiments. For instance, IKKα and IKKβ form a multi-subunit complex with NEMO [32] and can be used as controls in NEMO pull-down experiments (Fig. 5D).

To be sure that specific ubiquitin chain types have been completely depleted from the cell extracts, the supernatants from the first pull-down should be subjected to a second pull-down (Fig. 5E). Such optimisation studies should be performed at the start of each new project to determine the amount of immobilised UBD-coupled beads that need to be added to a given amount of cell extract to completely deplete the pUb-POI and/or pUb chain of interest.

The UBD pull-down method is particularly powerful for selectively enriching the ubiquitylated forms of proteins that are only ubiquitylated to a low stoichiometry in cells. For example, the ubiquitylated form of RIP1 could only be detected after capture on immobilised NEMO, and was virtually undetectable in cell extracts (Fig. 5F).

The immobilised UBDs, but not ubiquitin-binding-defective mutants of the UBD, will also capture proteins that bind non-covalently to the ubiquitin chains captured by the UBD. These proteins can be identified by mass spectrometry followed by immunoblotting [4].

UBDs can also be used in “Far Western” blotting experiments to detect pUb chains on membranes. In this procedure, protein samples separated by SDS-PAGE are transferred to a membrane and incubated with the appropriate purified UBD (normally coupled to biotin or horse radish peroxidase). A suitable detection system is then used to identify the bound UBD and hence the pUb chains with which it interacts [33]. An advantage of this technique is that it does not normally require sample denaturation in solution or on membranes (see Section 4.1). In fact, denatured ubiquitin chains and ubiquitylated proteins may not interact with their cognate UBDs, resulting in failure to identify an interaction. In this procedure, it is important to use ubiquitin-binding-defective mutants of UBDs (Table 1) in parallel with wild type UBDs to exclude ubiquitin-independent interactions with other UBD-binding partners.

**Box 1. Capturing pUb chains by using UBDs.**

**Advantages:**

**Fig. 6:**
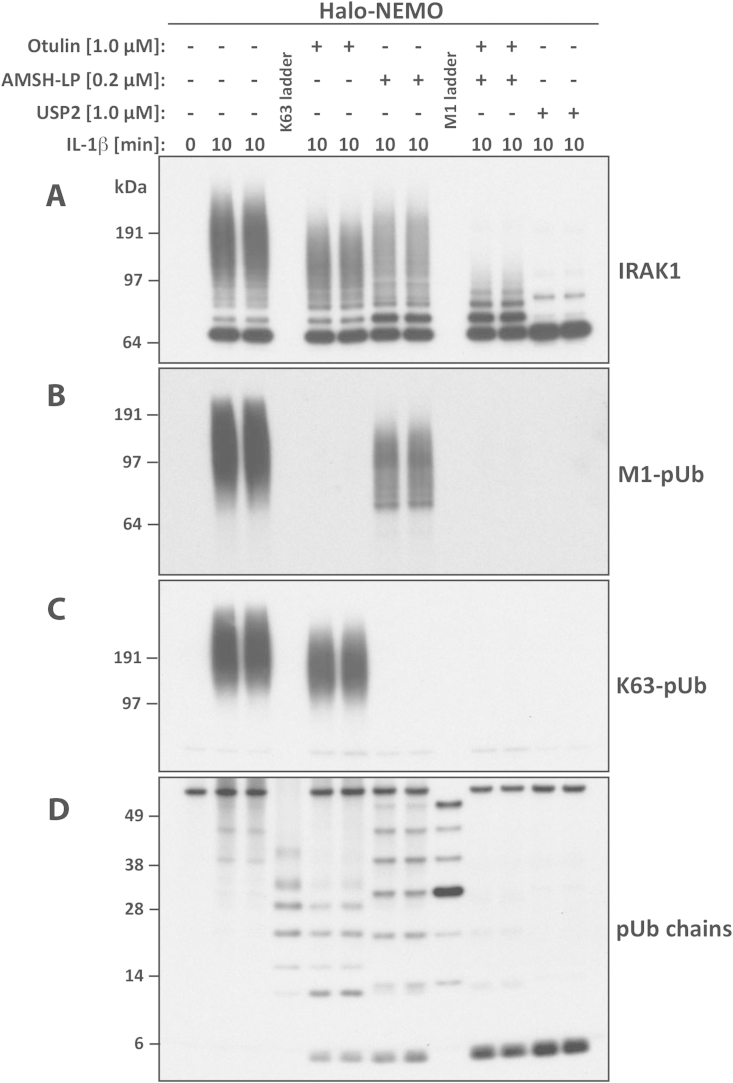
IL-1 induces the formation of K63/M1-pUb hybrid chains. IL-1R cells were stimulated for 10 min with 5 ng/ml IL-1β, lysed (see Supplementary section 1.3) and pUb chains as well as ubiquitylated proteins were captured from the cell extracts using immobilised Halo-NEMO (see Supplementary Section 1.4). Prior to SDS-PAGE, samples were incubated with λ-PPase (100 Units) in the absence or presence of AMSH-LP, Otulin, AMSH-LP plus Otulin, or USP2 (concentrations indicated). Following SDS-PAGE and transfer to PVDF membranes, immunoblotting was carried out with antibodies that recognise IRAK1 (panel A), M1-pUb chains (panel B), K63-pUb chains (panel C) or all types of ubiquitin chains (panel D). K63-pUb (4 ng) and M1-pUb (10 ng) oligomeric markers (Supplementary Section 1.1), which are shown in lanes 4 and 9, were used as standards to identify the small ubiquitin oligomers generated by treatment with the different DUBs ​(Section 6.2).

• UBD pull-down assays permit the simultaneous isolation of the ubiquitylated forms of many endogenous proteins from cell extracts without any need to overexpress ubiquitin.
• Linking multiple UBDs together provides an opportunity to increase avidity and affinity for poly-ubiquitin chains. Different systems, in particular the MultiDsk or TUBEs constructs, are now available commercially and well-characterised [28], [29].
• Tag-systems based on the covalent coupling between a protein fusion tag and synthetic chemical ligands attached to a solid support (e.g. the HaloTag system) allow for stringent and extensive washing after the pull-down and minimise non-specific binding.
• An important benefit of the pull-down method is the absence of antibody heavy and light chains, which can otherwise interfere with analysis by immunoblotting.
• Recombinant TUBEs or MultiDsks have also been shown to protect pUb chains and ubiquitylated proteins in cell extracts from hydrolysis by a variety of DUBs [28], [29]. This is another way to circumvent the need for chemical inhibition of DUBs after cell lysis to prevent deubiquitylation (see Section 2.1).
• Ubiquitin-binding-defective mutants can be included as controls. These mutants (Table 2 and Fig. 6A) enable proteins that interact with the UBD in a ubiquitin-dependent manner to be identified very easily and distinguished from those that bind in an ubiquitin-independent manner.

**Table 2:** Specificity of DUBs useful for ubiquitin chain architecture analysis.

DUB	Specificity	Reference
USP2	All pUb chains and mono-ubiquitylated proteins	[31]
AMSH-LP	K63	[18]
OTULIN	M1	[38]
OTUB1	K48	[39]
USP5 (IsoT)	Free pUb chains not attached to any other protein	[36]
vOTU	All pUb chains except M1, K27 and K29	[31]

**Points to consider:**

• Glutathione S-transferase (GST) forms dimers and the dimeric GST moiety of GST-fusion protein can bring together two UBA (ubiquitin-associated) domains in a configuration that have been reported to alter their linkage selectivity [34]. The length of the ubiquitin chains and their affinity for the UBD may also be modified by GST dimerization. For these reasons it is recommended that GST-tagged UBDs are not used for studies of this type.
• Although some UBDs have >100 fold higher affinity for some Ub chain types than others [21], [22], the UBD may still pull down Ub-chain types with which it interacts more weakly, particularly if they are expressed at high levels.

## 6 Use of deubiquitylases to identify ubiquitin chain linkage type and topology

### 6.1 Chain-type specific DUBs

Over the past few years DUBs with characteristic specificities for particular chain types have been identified [31], [35]. The exploitation of these linkage-specific DUBs provides a simple and powerful way to identify and distinguish between the different types of pUb chains that are attached to ubiquitylated POIs. This approach requires that the POI or pUb chains are first captured from cell extracts (see Section 5) prior to the DUB treatment and analysis of the pUb chain pattern by immunoblotting (Fig. 6).

At present, several DUBs have been identified that have a sufficiently high specificity for the hydrolysis of just one type of Ub linkage (Table 2). However, the non-specific DUB, termed IsoT or USP5 can be used to identify the proportion of free ubiquitin chains in a cell extract that are not attached covalently to any other protein. This is because IsoT can hydrolyse every type of ubiquitin linkage, but cannot cleave ubiquitin chains that are attached via their C-terminus to other proteins, termed “anchored” ubiquitin chains [36], [37]. In contrast, USP2 can hydrolyse every type of ubiquitin chain, whether they are free or anchored (Table 2) and is therefore useful for reconverting ubiquitylated proteins to their unmodified forms (Fig. 5B and C).

Cells contain several Ub-like proteins that can be attached covalently to proteins by enzymatic reactions similar to those that attach ubiquitin to proteins. These include SUMO, NEDD8 and ISG15. Although some DUBs, such as USP2 and IsoT/USP5 have been reported to interact with both ubiquitin and ISG15 using active-site directed probes [40], these DUBs failed to cleave ISG15 from ISGylated proteins when tested experimentally [41]. USP21 is the only USP so far shown to cleave ISG15 as efficiently as ubiquitin from proteins [42], [43]. To date, no DUB has been reported to cleave SUMO linkages and only the DUB termed UCH-L3 has been shown to cleave the NEDD8 conjugated to proteins when tested *in vitro* [44]. UCH-L3 might therefore function as a C-terminal hydrolase for both NEDD8 and ubiquitin in cells.

NEDD8-specific proteases, such as SENP8/NEDP1 [45], [46] or SUMO-specific proteases, such as SENP2 (R. Hay, personal communication), can however be used to discriminate between ubiquitylation, NEDDylation and SUMOylation, These proteases may be valuable for detecting these Ub-like modifications specifically when they are attached to the POI or their presence in hybrids containing polySUMO chains attached covalently to ubiquitin chains [47], [48], as discussed in Section 6.2.

### 6.2 Detection of hybrid ubiquitin chains

Heterotypic pUb chains of complex topology (termed hybrid chains, Fig. 1) have recently been identified and serve specialised signalling functions within the cell [4], [5], [6]. Direct evidence for the formation of K63/M1-pUb hybrids (Fig. 6A–D) came from the finding that when the pUb chains formed in response to cell stimulation with IL-1 [4] were captured by Halo-NEMO (see Section 5) and then incubated with Otulin to hydrolyse M1-pUb chains specifically (Table 1), small K63-pUb oligomers were liberated that could be detected by immunoblotting (Fig. 6D). In line with this, Otulin-treatment also reduced the size of the large K63-pUb chains induced after IL-1 stimulation (Fig. 6C). Conversely, when the same pUb chains were incubated with AMSH-LP, a DUB that hydrolyses K63-pUb chains specifically (Table 1), the size and amount of the large M1-Ub chains was reduced (Fig. 6B) and small faster-migrating M1-pUb oligomers were released from K63/M1-pUb hybrid chains (Fig. 6D). In these experiments the small K63-Ub and M1-Ub oligomers could be distinguished from one another and identified by their characteristic and distinct electrophoretic mobilities during SDS-PAGE (see Section 4.3). Finally, treatment with Otulin increased the electrophoretic mobility of polyubiquitylated IRAK1 but the polyubiquitin chains remaining attached to IRAK1 were much larger than the monoubiquitylated forms of IRAK1 (Fig. 6A) [4]. This and other evidence indicated that the M1-pUb chains were attached covalently to pre-formed K63-Ub chains attached to IRAK1. Analogous results were obtained when ubiquitylated RIP2 formed upon cell stimulation with muramyl dipeptide (a component of bacterial peptidoglycans) was treated with Otulin [49], demonstrating that hybrid chains containing M1-Ub and at least one other ubiquitin chain linkage type are commonly formed when innate immune signalling networks are activated [4], [49].

The presence of hybrid ubiquitin chains in other cell signalling networks can be analysed in similar ways. For example, small K29-linked pUb oligomers were released from the pUb chains captured by immobilised tandem NZF domains of TRABID (Table 1), following incubation with vOTU, a DUB that does not hydrolyse K29 linkages (Table 2). This indicates that K29 linkages are present in cells as part of hybrid ubiquitin chains consisting of at least two different linkage types [5].

Hence, a UBD that binds to one type of ubiquitin chain specifically may nevertheless capture other types of ubiquitin linkage because proteins can be modified at multiple lysine residues with different types of ubiquitin chains and/or because a ubiquitin chain may contain more than one type of linkage. A protein may also be a component of a multi-protein complex, and the different protein components of the complex may be modified by different types of ubiquitin chains (or may not be ubiquitylated at all). Thus the UBD pull-down method can reveal a considerable amount of interesting information about the composition and ubiquitylation of protein complexes.

## 7 Immunoprecipitation of ubiquitin chains and ubiquitylated proteins

### 7.1 Immunoprecipitation of endogenous, ubiquitylated POIs

Instead of capturing ubiquitylated proteins on immobilised UBDs, the endogenous ubiquitylated proteins can be immunoprecipitated from cell extracts using POI-specific antibodies that have been coupled to a solid support (e.g. Protein G agarose), followed by immunoblotting with a different antibody to the POI to see if it is ubiquitylated. The type of ubiquitin chain attached to the POI can then be analysed by treatment with deubiquitylases as outline in Section 6.

**Box 2. Immunoprecipitation of endogenous ubiquitylated POIs.**

**Advantages:**

• The endogenous POI is immunoprecipitated from the cell extracts without the need to overexpress exogenous ubiquitin.

**Potential pitfalls:**

• The immunoprecipitation of the POI has to be performed under non-denaturating conditions, i.e. it is not compatible with buffers containing high concentrations of SDS, urea or guanidinium chloride (see Section 8.1). Thus, proteins that interact with the POI will be co-immunoprecipitated. An anti-Ub antibody cannot therefore be used in subsequent immunoblotting experiments to determine the ubiquitylation status of the POI since it will also detect the ubiquitylation of interacting proteins and other proteins present as contaminants in the IPs.
• Ubiquitin chains attached to lysine residues within or near the antibody-binding region may prevent the isolation and capture of some or all forms of the ubiquitylated POI. These experiments should therefore be conducted using polyclonal antibodies raised against two different regions of the POI (see Section 7.3).
• Depending on the size of the POI, the presence of antibody heavy and light chains may interfere with the detection of the POI by immunoblotting. To avoid this problem, the antibody can first be cross-linked to a solid support, using agents such as dimethyl pimelidinate (DMP). However, this procedure may decrease the affinity of the antibody for its target protein.

### 7.2 Immunoprecipitation of endogenous ubiquitin

The procedure outlined in Section 7.1 can in theory be reversed and all the ubiquitylated proteins in the cell immunoprecipitated with anti-ubiquitin, followed by immunoblotting with an antibody to the POI to see if it is ubiquitylated. However, in practice, due to the high abundance of ubiquitin in cells (about 85 μM in HEK293 cells) [50], the amount of anti-ubiquitin antibody required to quantitatively immunoprecipitate all the ubiquitylated proteins from cell extracts makes this method impractical.

### 7.3 Use of monoclonal anti-POI antibodies to detect the ubiquitylated POI

**Fig. 7:**
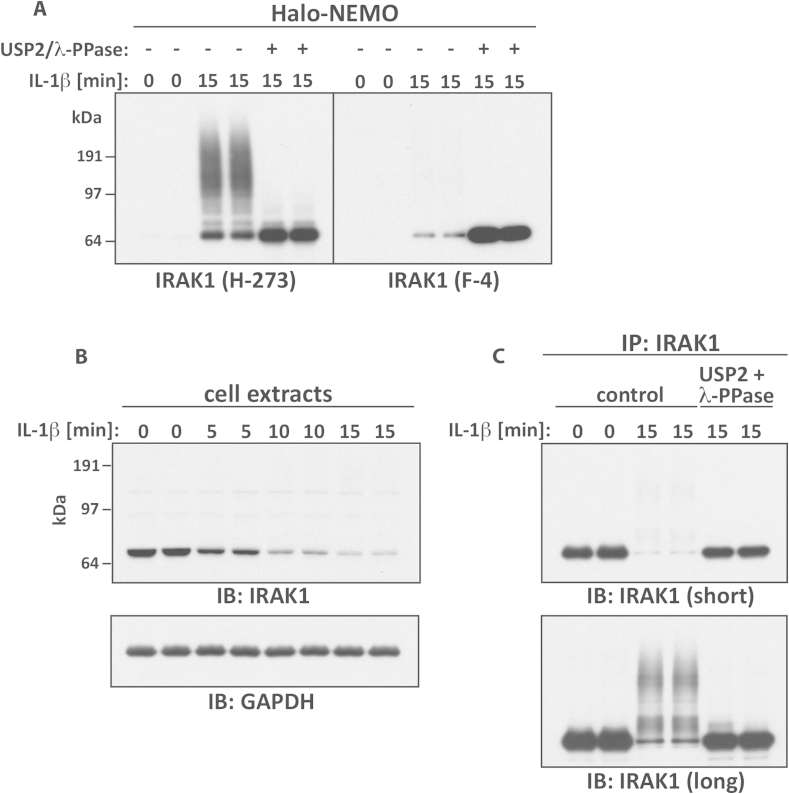
Blocking of antibody epitopes by ubiquitin chains. (A) The experiment was performed as in Fig. 5B, except that immunoblotting was carried out using two different anti-IRAK1 antibodies (polyclonal H-273 and monoclonal F-4). (B) As in A, but after stimulation with IL-1β for the times indicated, cell extracts (20 μg protein) were denatured in SDS, subjected to SDS-PAGE and immunoblotted with the antibodies shown. The antibody against GAPDH was used as a loading control. (C) As in B, except that IRAK1 was immunoprecipitated from the cell extracts (1 mg protein) and incubated without (control) or with USP2 plus λ-PPase. Samples were subjected to SDS-PAGE and IRAK1 was visualised by immunoblotting for 5 s (“short”) (upper panel) or 60 s (“long”) (lower panel) with an anti-IRAK1 antibody.

When using monoclonal antibodies against the POI, it is critical to remember that the antibody epitope might be blocked or masked by the presence of a ubiquitin chain, preventing the antibody from recognising the modified version of the POI. For example, a monoclonal IRAK1 antibody (F-4) only recognises the modified protein very weakly, in contrast to a polyclonal antibody against IRAK1 (H-273) (Fig. 7A). To minimise this problem, which can lead to the erroneous conclusion that a protein has been degraded and not ubiquitylated, different monoclonal antibodies raised against different epitopes or polyclonal antibodies should be tested.

### 7.4 Immunoprecipitation of POI-interacting proteins

The ubiquitylation state of the POI can also be studied by immunoprecipitation of a protein with which it interacts. For example, the interaction of TNF with the TNF receptor 1 (TNF-R1) induces the formation of a multi-protein complex, which includes the protein RIP1. The ubiquitylation of RIP1 can therefore be studied by stimulating cells with FLAF-tagged TNF, followed by immunoprecipitation of the TNF-R1 signalling complex with anti-FLAG antibodies and immunoblotting with anti-RIP1 (Fig. S3).

## 8 Overexpression of ubiquitin

### 8.1 Tagged ubiquitin

In this method, vector-generated ubiquitin, covalently linked to protein tags like hemagglutinin (HA), c-Myc, hexa-histidine (His_6_) or FLAG octapeptide are transfected into the cell of interest, and become attached to target proteins because they compete with the endogenous unmodified ubiquitin. An advantage of this method is that anti-tag affinity purification methods can be used to enrich ubiquitylated proteins of interest, which can then be identified by immunoblotting with antibodies to the POI. Alternatively, the POI can first be immunoprecipitated with a specific antibody and its ubiquitylation status analysed by immunoblotting with the anti-Tag antibodies. However, in these experiment the ubiquitylated species detected with the anti-tag antibody may not be the POI, but a POI-interacting protein or a contaminant. It is therefore critical to remove such proteins from the POI prior to immunoprecipitating with the anti-tag antibody. This is generally achieved by including SDS in the cell lysis buffer at a final concentration of 1%, and diluting the cell extract to 0.1% SDS before immunoprecipitating the POI. This procedure disrupts protein complexes held together by non-covalent forces. The dilution to 0.1% SDS and absence of thiols in the lysis buffer is critical to prevent the denaturation and inactivation of the immunoprecipitating antibody. However, the effectiveness of the dissociation procedure has to be controlled by immunoblotting with antibodies to proteins known to interact with the POI comparing samples before and after the addition of 1% SDS. It is also important to check that proteins do not re-associate when the SDS concentration is diluted to 0.1%. A serious disadvantage of this method is that the overexpression of tagged ubiquitin may lead to the ubiquitylation of proteins that are not normally ubiquitylated in cells. In addition, the presence of any tag attached to the N- or C-terminus of ubiquitin will prevent the formation of M1-linked ubiquitin chains [51].

**Box 3. Overexpression of tagged versions of ubiquitin.**

**Advantages:**

• When His_6_-tagged ubiquitin is used, ubiquitylated proteins can be isolated by Ni^2+^ IMAC (immobilised metal ion affinity chromatography). This method is compatible with strong denaturing conditions (e.g. SDS, 8M urea, 6M guanidinium chloride) that disrupt non-covalent protein–protein interactions and inhibit remaining DUB activities.
• Efficient and sensitive anti-tag antibodies are available commercially.

**Disadvantages:**

• Artificially overexpressing tagged versions of ubiquitin may affect the physiology and bioactivity of the ubiquitin molecule in ways that cannot be easily assessed, and lead to the ubiquitylation of proteins that are not normally ubiquitylated in cells.
• The presence of any tag linked to the N- or C-terminus of ubiquitin will prevent the formation of M1-linked Ub chains [51].

In humans, the identical ubiquitin molecule is encoded by four different genes (UBC, UBB, UBA52 and UBA80), the products of which are then processed to release free ubiquitin. A His_6_-tagged ubiquitin has been expressed under the endogenous UBC gene [52] to enable the tagged-ubiquitin to be expressed at physiological levels and so minimise potential problems associated with the overexpression of ubiquitin. However, reducing the level of expression of tagged ubiquitin may result in the endogenous wild-type ubiquitin being used preferentially for ubiquitin chain assembly. It is therefore recommended when adopting this procedure, that three of the endogenous genes are silenced at the same time that the 4th is replaced by a modified gene encoding the tagged ubiquitin [53], [54]. This was first achieved using RNAi, but might be done more efficiently in the future by CRISPR/CAS9 gene editing technology.

Given all the limitations of using tagged ubiquitin, we recommend that the Method described in Section 5 is used instead.

### 8.2 The overexpression of ubiquitin mutants

In this procedure all but one of the lysine residues in ubiquitin are mutated to arginine to prevent their ubiquitylation and N- or C-terminally tagged versions of these mutants are used to study the formation of one type of ubiquitin chain in different cellular contexts. This approach suffers from the same disadvantages discussed in Section 8.1. In addition, there is no guarantee that any of these ubiquitin mutants will be recognised by E1, E2 and E3 ligases as efficiently as the wild type endogenous ubiquitin, that they are fully functional, expressed at the same levels in cells and that the observed effects are not due to the mutants being folded improperly. Moreover the incorporation of the mutant ubiquitin into pUb chains may affect their affinities for UBDs and affect the rate at which they are hydrolysed by DUBs [55], [56], [57]. These mutants can be useful for generating specific types of ubiquitin linkages *in vitro*, but their use in overexpression studies is not recommended.

## 9 Determination of the stoichiometry of protein ubiquitylation

### 9.1 Determining the ratio between unmodified and ubiquitylated proteins

To estimate the proportion of ubiquitylated to unmodified protein it is usually necessary to immunoprecipitate the POI, followed by immunoblotting with an antibody that recognises the POI specifically. This ensures the simultaneous detection of unmodified, mono-ubiquitylated and/or poly-ubiquitylated species of the POI. However, frequently, only a tiny proportion of the POI undergoes ubiquitylation in cells (e.g. Fig. 5F). Nevertheless, in the case of RIP1, this low level of ubiquitylation appears to be sufficient to drive the TNF signalling network [24]. Therefore trace ubiquitylation of proteins can have important consequences for cell function.

### 9.2 Apparent disappearance of proteins from cells

The rapid disappearance of proteins from cells in response to an extracellular signal, is frequently assumed to be caused by ubiquitylation followed by proteasomal degradation. However, non-degradative poly-ubiquitylation events can convert the unmodified protein to a great variety of slowly migrating species (see Section 10), which can sometimes be difficult to detected by immunoblotting because any one ubiquitylated species is present at such a low concentration compared to the unmodified protein (Fig. 5F). This problem is compounded if the anti-POI antibodies are unable to recognise the ubiquitylated forms of the protein (Fig. 7A) and lead to the incorrect inference that the protein has disappeared from the cell. To determine whether failure to detect a protein by immunoblotting is caused by its disappearance or by conversion to many ubiquitylated, phosphorylated and other covalently modified forms of the protein, it is necessary to treat the protein with a broad specificity DUB and a broad-spectrum protein phosphatase to completely remove these covalent modifications. In the case of IRAK1, the “disappearance” of the protein from cell extracts (Fig. 7B, left panel) could be fully reversed when immunoprecipitated IRAK1 was treated with USP2 plus phage λ-phosphatase (λ-PPase) (Fig. 7B, right panel). Thus, earlier studies that had concluded that IRAK1 is rapidly degraded by the proteasome were erroneous [58].

## 10 Appearance of pUb chains and polyubiquitylated proteins in immunoblotting experiments

It will be apparent from reading this article that small ubiquitin oligomers of a particular chain type appear on SDS-PAGE as discrete bands, separated by intervals of about 8 kDa, i.e. the molecular mass of ubiquitin (Fig. 2, Fig. 3C). In theory, when a substrate protein is modified with a homotypic pUb chain it should also be visualised as a ladder of bands extending upwards from the expected location of the unmodified protein in the gel. However, in practice, ubiquitylated proteins are normally seen as a “smear” of ubiquitylated material extending upwards from the POI to the top of the gel, which can be detected by immunoblotting with antibodies raised against the protein of interest or against ubiquitin. This smear, as opposed to a ladder, is caused by heterogeneity of the modification, which could be due to the presence of more than one type of ubiquitin linkage, a mixture of polyubiquitylation and multi-monoubiquitylation or a combination of ubiquitylation and other modifications such as phosphorylation and sumoylation. The presence of hybrid pUb chains will also result in a smear at the level of immunoblotting (Fig. 1A). A major technical challenge for the future is to find a simple immunoblotting method for distinguishing between polyubiquitylation and multi-monoubiquitylation (Fig. 1A).

## 11 Summary of recommendations

In the preceding sections we have discussed the advantages and disadvantages of the procedures for the detection of ubiquitin chains and ubiquitylated proteins by immunoblotting that are in current use and how they are best exploited to obtain accurate, reliable and reproducible data. In this section we briefly summarise our main recommendations.

**Recommendation 1** (Section 2.1): Use IAA or NEM in the cell lysis buffer at the correct concentration needed to completely prevent deubiquitylation of the POI and/or the polyubiquitin chains of interest.

**Recommendation 2** (Section 2.2): Block the catalytic activity of the Proteasome by treating cells for 1 h with 25 μM MG132 to induce the accumulation of the ubiquitylated form of the POI.

**Recommendation 3** (Section 3.1): Choose the most appropriate polyacrylamide gel and buffer system for the separation of pUb chains and ubiquitylated-POI, depending on the length of the pUb chains and the size of the unmodified POI.

**Recommendation 4** (Section 3.1): If a gel of a single polyacrylamide concentration is used, check that the longest pUb chains have really entered the separating gel. The stacking gel should therefore not be removed, but also submitted to transfer to membranes prior to immunoblotting.

**Recommendation 5** (Section 3.1): Since ubiquitylation of the POI can cause it to migrate over a very wide range of molecular mass, the PVDF or NC membrane should not be cropped prior to immunoblotting.

**Recommendation 6** (Section 3.2): If you are struggling with signal strength after transferring the gel to NC membranes, transfer to PVDF membranes instead, especially for the detection of short ubiquitin chains.

**Recommendation 7** (Section 4.1): Expose membranes to denaturing conditions to enhance the binding of anti-ubiquitin antibodies to ubiquitin chains, particularly if the antibody-binding site is buried within the intact ubiquitin protein and therefore not exposed after transfer to membranes. This procedure might not be necessary for every anti-ubiquitin antibody, but should be tested whenever antibodies are used for the first time.

**Recommendation 8** (Section 4.2): It is essential to analyse and validate the performance of ubiquitin antibodies carefully in terms of pUb chain-type affinity and preference before deciding which one to use. Insufficient attention has been paid to potential antibody bias and preferences in the past and may require re-examination of a number of published studies. One should not assume that different batches of antibody obtained from the same supplier behave identically.

**Recommendation 9** (Section 4.3): When using pUb chain-specific antibodies, their performance should be checked with pure ubiquitin oligomers of known linkage type alongside the test samples. This ensures that specificity of the antibody is maintained throughout the experiment (primary antibody dilution, IP conditions, use of PVDF or NC membranes).

**Recommendation 10** (Section 5): To detect ubiquitin modifications of POIs, use immobilised UBDs to capture them, followed by immunoblotting with anti-POI antibodies.

**Recommendation 11** (Section 5): Use non-specific DUBs, like USP2, to prove that the observed modification is due to the attachment of ubiquitin chains and not to another type of modification, including modification by other ubiquitin-like modifiers (Nedd8, SUMO, ISG). Use IsoT to examine whether ubiquitin chains are “free” or are attached covalently to another protein(s).

**Recommendation 12** (Section 5): Use Ubiquitin-binding-defective mutants of UBDs to establish that the interaction of a protein with a UBD is ubiquitin-dependent.

**Recommendation 13** (Section 5): At the outset of each study, determine the amount of UBD-coupled beads required to completely deplete the ubiquitin chains of interest and/or the ubiquitylated form of the POI from the cell extracts (for further technical details see the Supplementary Information).

**Recommendation 14** (Section 6.1): When using DUBs, their specificity should be checked using recombinant ubiquitin oligomers of different chain-type and length alongside the test samples. Although AMSH-LP hydrolyses K63-Ub chains and not other type of ubiquitin chain, at high concentrations it can also hydrolyse the isopeptide bond formed between the C-terminus of ubiquitin and the ε-amino group of a lysine residue in the POI to which the pUb chain is attached. Samples should therefore be treated for the minimum length of time and at the lowest possible concentration of AMSH-LP needed to hydrolyse all the K63-Ub chains in the sample (see Supplementary Information for further technical details).

**Recommendation 15** (Section 7): As for all immunoprecipitation/immunoblotting studies, the optimal amount and concentration of the antibodies to use, and the time needed to ensure efficient immunoprecipitation should be determined at the outset of the study.

**Recommendation 16** (Section 7.1): When the POI has been immunoprecipitated, do not use anti-ubiquitin antibodies to analyse whether it is ubiquitylated. This is because the anti-ubiquitin antibodies will also detect ubiquitylation forms of proteins that interact with the POI or that were immunoprecipitated non-specifically by the antibody. Always use anti-POI antibodies instead as a requirement to establish that the POI is modified by ubiquitin chains.

**Recommendation 17** (Section 7.3): Remember that some antibodies may only recognise the unmodified protein and not the ubiquitylated protein. Different antibodies raised against different epitopes, or polyclonal antibodies should also be used to circumvent this problem.

**Recommendation 18** (Section 8): The overexpression of tagged-versions of wild type and mutant ubiquitins may lead to the abnormal ubiquitylation of proteins and to erroneous conclusions being reached, and is not recommended for reasons discussed in Sections 8.1 and 8.2. However, if such experiments are performed, the anti-tag antibodies should only be used for immunoprecipitation and anti-POI antibodies should be employed to analyse the ubiquitylation state of the POI (see Recommendation **16**).

**Recommendation 19** (Section 9.2): The immunoprecipitated POI should always be incubated with and without USP2 to hydrolyse all the ubiquitin chains, prior to immunoblotting with antibodies to the POI. These experiments enable the proportion of the POI that is ubiquitylated to be assessed and can be used to determine whether the POI has been ubiquitylated or degraded.

## Author contribution

Christoph Emmerich and Philip Cohen designed, and Christoph Emmerich performed the experiments. Philip Cohen and Christoph Emmerich wrote the paper.

## Declaration of interest

The authors declare no conflict of interest.

## Funding

The work was supported by the Wellcome Trust [WT100294], the UK Medical Research Council [MRC-MR/K000985/1], AstraZeneca, Boehringer Ingelheim, GlaxoSmithKline, Janssen Pharmaceutica, Merck-Serono and Pfizer.

## Figures and Tables

**Figure:**
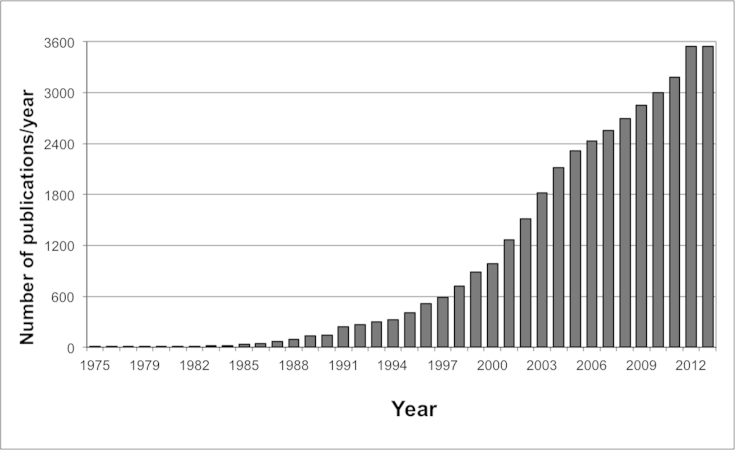


**Figure:**
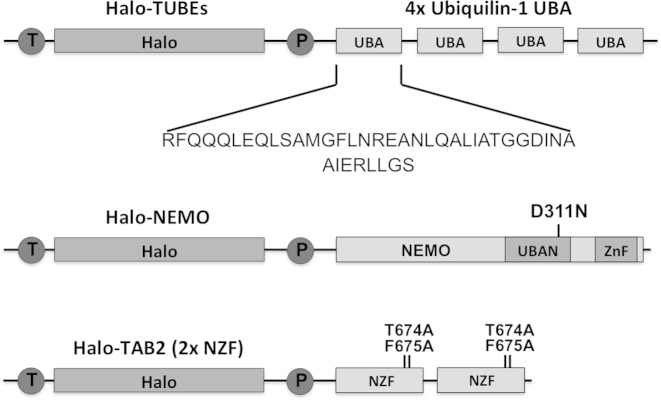


**Figure:**