# Supplementary material for: Optimising methods for the preservation, capture and identification of ubiquitin chains and ubiquitylated proteins by immunoblotting
Source: Biochem Biophys Res Commun. 2015 Oct 9;466(1):1–14. doi: 10.1016/j.bbrc.2015.08.109 (PMC4709362; doi:10.1016/j.bbrc.2015.08.109)
Supplement: Supplementary file 1 [file mmc1.doc]

**Supplementary information**

**1 Material and Methods**

**1.1 Material and Proteins**

MG132 was purchased from Calbiochem (#474790) and Iodoacetamide from Sigma (#I6125). The HaloLink™ Resin and pFN18A HaloTag® T7 Flexi® Vector were obtained from Promega. Vectors expressing Halo-tagged NEMO, NEMO[D311N] and TUBEs were generated by the DNA cloning team, Division of Signal Transduction Therapy (DSTT), MRC Protein Phosphorylation and Ubiquitylation Unit (MRC-PPU), University of Dundee, UK. IL-1β and TNF were expressed as GST-fusion proteins in *E.coli*, cleaved with PreScission protease to release IL-1β[117-268] and TNF[77-234], respectively and purified by gel-filtration on Superdex G200. FLAG-tagged TNF (#ALX-522-008) was purchased from Enzo Life Sciences. The ubiquitin-like modifier activating enzyme (UBE1), the E2 ubiquitin-conjugating enzyme UBE2L3 (also called UbcH7), the deubiquitylases rat USP2 (catalytic domain residues 259-605), AMSH-LP (catalytic domain residues 264-436) full-length Otulin and TEV-protease [S219N] were expressed and purified by the Protein Production Teams of the DSTT and MRC-PPU, University of Dundee UK. The K48-linked (K48_2-7_), K63-linked (K63_2-7_) and M1-linked (M1_2-7_) ubiquitin oligomers were purchased from Boston Biochem and bacteriophage λ protein phosphatase (λ-PPase) from New England Biolabs (NEB).

**1.2 Antibodies**

Antibodies against GAPDH (#2118), Ubiquitin (#3936) and IκBα (#4814) were obtained from Cell Signaling Technology; against K63-pUb (#05-1313), K11-pUb (#MABS107), K48-pUb (#05-1307) and IKKβ from Millipore and against IRAK1 (#sc-7883 and #sc-5288) and TNF-R1 (#sc-8436) from Santa Cruz. Antibodies against RIP-1 (#610459) were from BD Biosciences, against ubiquitin (#Z0458) from Dako and against ubiquitin (#BML-PW8805 and #BML-PW8810) from Enzo Life Sciences. Anti-FLAG M2 affinity gel (#A2220) was purchased from Sigma. The M1-pUb antibody has been described before [1]. The IRAK1 (sheep number S690C, third bleed) and HOIP (sheep number S174D, third bleed) antibodies for immunoprecipitation experiments were produced by the Antibody Production Team of the DSTT, MRC-PPU, University of Dundee.

**1.3 Cell stimulation and Cell lysis**

Human HEK293 cells stably overexpressing the IL-1 receptor [2] or human THP-1 cells were stimulated with 5 ng/ml IL-1β or 10 ng/ml TNF, respectively, for the times indicated in the figure legends. Cells were rinsed in ice-cold PBS and lysed (50 mM Tris/HCl pH 7.5, 1 mM EGTA, 1 mM EDTA, 1% (v/v) Triton X-100, 1 mM sodium ortho-vanadate, 50 mM NaF, 5 mM sodium pyrophosphate, 0.27 M sucrose, 10 mM sodium 2-glycerophosphate, 0.2 mM phenylmethyl-sulphonyl fluoride, 1 mM benzamidine) for 15-30 min at 4°C. In addition, 100 mM Iodoacetamide was added to inactivate deubiquitylase activities, unless stated otherwise. Cell lysates were clarified by centrifugation at 14,000 x g for 30 min at 4°C. The Bradford protein assay was used to measure the protein concentrations of cell extracts.

**1.4 Coupling of Halo-tagged proteins and pull-down assays**

Halo-NEMO, the poly-ubiquitin binding-defective mutant Halo-NEMO[D311N] and Halo-TUBEs (Figure S2) were expressed in *E. coli* and Halo-tag fusion protein expression was induced with 200 µM Isopropyl-β-D-thiogalactoside (IPTG). The bacteria were lysed in 50 mM Tris/HCl pH 7.5, 150 mM NaCl, 1 mM EGTA, 1 mM EDTA, 0.1% 2-mercaptoethanol, 1 mM benzamidine, 0.2 mM phenylmethyl-sulphonyl fluoride (PMSF) and sonicated. To couple Halo-tagged proteins to the HaloLink resin, beads were added at a ratio of 1 ml of resin per 10 ml of cleared lysate and incubated over night at 4°C as described by the manufacturer. The resin was then washed extensively with 50 mM Tris/HCl pH 7.5, 0.5 M NaCl, 0.1 mM EDTA, 0.27 M sucrose, 0.03% (w/v) Brij-35, 0.1% (v/v) 2-mercaptoethanol, 0.2 mM PMSF and 1 mM benzamidine and stored at 4°C. The coupling efficiency was controlled by re-suspending 5 µl of the resin in 50 mM Tris/HCl pH 8.0, 150 mM NaCl, 0.5 mM EDTA, 1 mM DTT containing 0.1 mg/ml TEV protease to release covalently bound proteins followed by SDS-PAGE and staining with Coomassie Blue. To capture pUb chains or ubiquitylated proteins, 2 mg (IL-1R cells) or 3 mg (THP-1 cells) of cell extract protein was incubated overnight at 4°C with 30 µl of Halo-NEMO, Halo-NEMO[D311N] or Halo-TUBEs beads, unless stated otherwise. The beads were washed three times with 1 ml of lysis buffer containing 500 mM NaCl and once with 1 ml of 10 mM Tris/HCl pH 8.0. The captured proteins were either treated as described in **Section 1.6** or released by denaturation in SDS and analysed by immunoblotting.

**1.5 Immunoprecipitation of IRAK1**

2 µg anti-IRAK1 (S690C) was pre-coupled to Protein G-Sepharose for 2 h at 4°C, unbound antibody was washed away and the antibody-coupled beads were used to precipitate IRAK1 from 1 mg of cell extract protein by incubating for 2 h at 4°C. Immunoprecipitated IRAK1 was treated with or without USP2 and phage λ phosphatase as described in **Section 1.6**.

**1.6 DUB-Assay**

Immunoprecipitated proteins or proteins captured by Halo-NEMO/Halo-TUBEs were washed three times with lysis buffer containing 0.5 M NaCl and once with 1 ml of 50 mM Tris/HCl pH 7.5, 50 mM NaCl, 5 mM DTT. Beads were then treated for 1 h at 37°C with USP2 (1.0 μM), AMSH-LP (0.1 µM), Otulin (1.0 μM) or λ-PPase (100 units/reaction) in 30 μl of 50 mM HEPES pH 7.5, 100 mM NaCl, 2 mM DTT, 1 mM MnCl_2_, 0.01% (w/v) Brij-35). The reaction was stopped by addition of LDS sample buffer and analysed by immunoblotting with the appropriate antibodies.

**1.7 Precipitation of the TNF Receptor Signalling Complex (RSC)**

For TNF-RSC analysis, THP-1 cells were either stimulated with 10 ng/mL FLAG-tagged TNF or not stimulated. Cells were rinsed in ice-cold PBS and lysed. Cell lysates were centrifuged at 15,000 x g for 30 min at 4°C and 100 ng FLAG-TNF was added only to the non-stimulated control extracts. FLAG-TNF was precipitated from 2 mg of cell extract protein by incubation for 16 h using M2 beads (Sigma). The beads were washed 3 times with 50 mM Tris/HCl pH 7.5, 1% (v/v) Triton X-100 and 250 mM NaCl and released with 1% (w/v) SDS. The TNF-RSC were analysed by immunoblotting using the antibodies indicated (**Figure S3**).

**1.8 In vitro ubiquitylation assay**

LUBAC was immunoprecipiated from 1 mg of IL-1R cell extract (cells lysed in the absence of Iodoacetamide) by adding 1 µg of anti-HOIP and 10 µl packed Protein G-Sepharose beads and incubating over night at 4°C. The beads were washed three times with 50 mM Tris/HCl pH 7.5, 1% (v/v) Triton X-100, 0.05% (v/v) 2-mercaptoethanol and 0.2 M NaCl and once with 50 mM Tris/HCl pH 7.5, 5 mM MgCl_2_. The ubiquitylation assay was started by resuspending the beads in 30 μl of 50 mM Tris/HCl pH 7.5, 2 mM DTT, 0.1 µM UBE1, 0.4 µM UbcH7, 10 µM ubiquitin, 5 mM MgCl_2_ and 2 mM ATP. After incubation for 1 h at 30°C, the reaction was stopped by denaturation in SDS. The M1-pUb chains generated were used to compare different SDS-PAGE gel systems (**Figure 3A**).

**1.9 SDS-PAGE running conditions**

The electrophoretic separation of proteins was carried out at a constant voltage of 125 V for 10 min and subsequently 185 V for around 60 min.

**1.10 Immunoblotting conditions**

For transferring proteins from SDS-PAGE gels onto nitrocellulose (pore size: 0.45 µm; capacity: 115-125 IgG/cm^2^; Amersham/ GE Healthcare) or PVDF (pore size: 0.45 µm; capacity: 294 IgG/cm^2^; Millipore) a maximum voltage of 30 V (130 mA) per gel was applied for 2.5 h (semi-dry transfer system). PVDF membranes were equilibrated in methanol for 20 sec prior to use. Afterwards, membranes were incubated for 1 h at ambient temperature with 5% (w/v) non-fat dry milk in Tris-buffered saline (TBS) buffer (25 mM Tris/HCl, pH 7.5, 0.15 M NaCl) with 0.25% (v/v) Tween-20 (TBST) to occupy non-specific protein-binding sites. Subsequently, membranes were subjected to immunoblotting with primary antibodies (diluted in 2% BSA/TBST) and secondary horseradish-peroxidase (HRP)-conjugated antibodies in 5% non-fat dry milk/TBST. Proteins were visualized using chemiluminescence detection systems (Amersham/ GE Healthcare and Millipore).

**2 Supplementary Figure legends**

**Figure S1:** Journal articles containing the word 'ubiquitin’ in the title, abstract or keywords between 1974 and 2013. Source: PubMed.gov

**Figure S2:** **Schematic representation of the Halo-TUBEs, Halo-NEMO and Halo-TAB2 constructs.** The UBA domain of Ubiquilin-1 was repeated in tandem 4 times (UBA units were separated by a short 8 amino acid spacer). The UBA sequences of Ubiquilin-1, full-length NEMO or the NZF domains of TAB2 were cloned into the pFN18A HaloTag expression vector generating Halo-TUBEs, HALO-NEMO or Halo-TAB2, respectively. The relevant protein sequence of the Ubiquilin’s UBA domain is indicated. T: TEV protease cleavage site; P: PreScission protease cleavage site.

**Figure S3: RIP1 is recruited to the TNFR signaling complex and modified in response to TNF.** HeLa cells (2 mg of cell extract protein) were left untreated or treated with FLAG-tagged TNF (50 ng/mL) for the times indicated. After cell lysis, 100 ng of FLAG-TNF was added to the unstimulated samples and FLAG-TNF was precipitated using anti-FLAG beads. The isolated proteins were analysed by immunoblotting with the indicated antibodies.

**3 References**

1. Matsumoto, M.L., et al., *Engineering and structural characterization of a linear polyubiquitin-specific antibody.* J Mol Biol, 2012. **418**(3-4): p. 134-44.

2. Li, X., et al., *Mutant cells that do not respond to interleukin-1 (IL-1) reveal a novel role for IL-1 receptor-associated kinase.* Mol Cell Biol, 1999. **19**(7): p. 4643-52.
